# Supplementary material for: Early-life exercise primes the murine neural epigenome to facilitate gene expression and hippocampal memory consolidation
Source: Commun Biol. 2023 Jan 7;6:18. doi: 10.1038/s42003-022-04393-7 (PMC9825372; doi:10.1038/s42003-022-04393-7)
Supplement: Supplementary file 2 — Supplementary Information [file 42003_2022_4393_MOESM2_ESM.pdf]

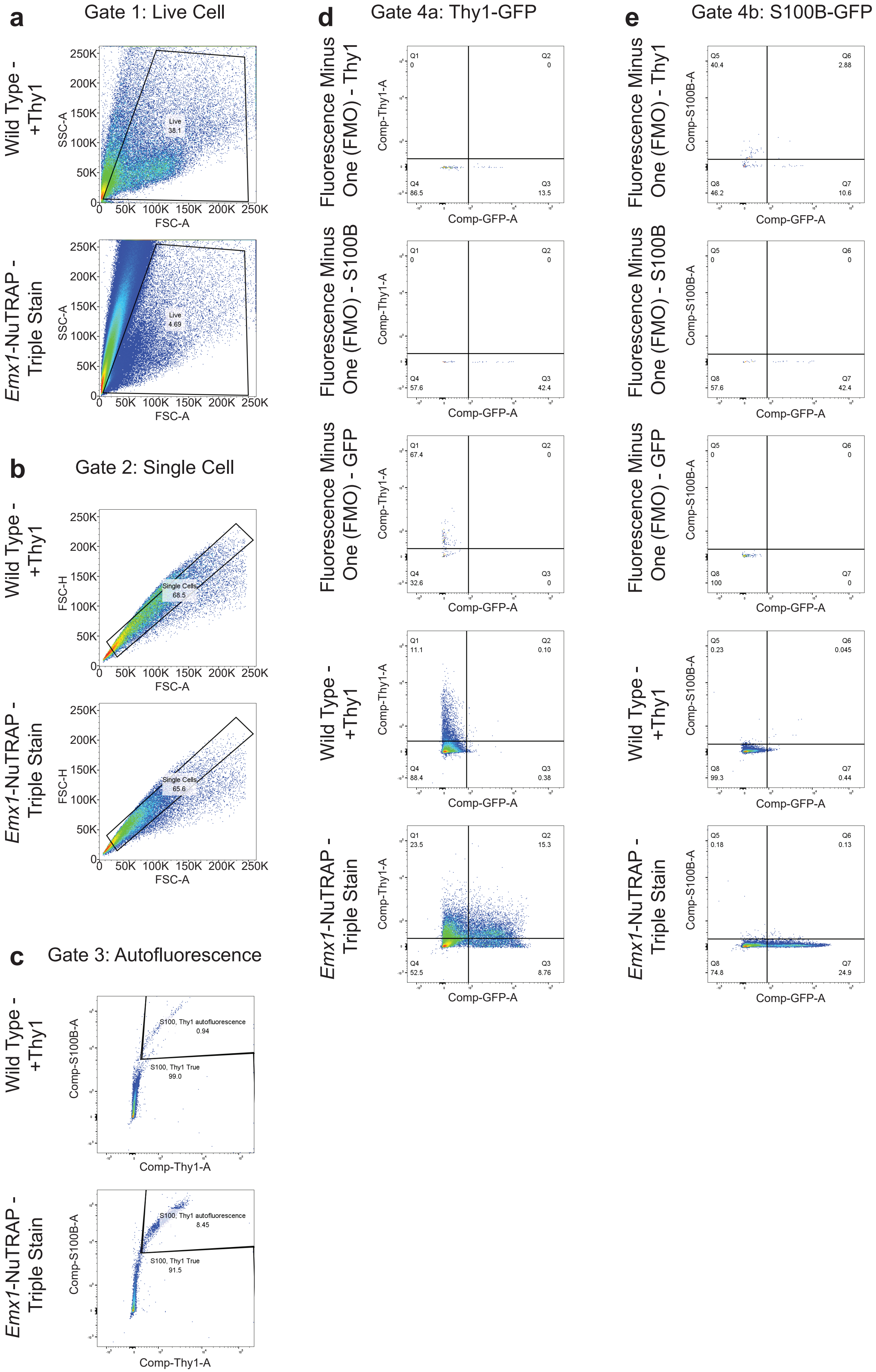
a
Gate 1: Live Cell
Wild Type - +Thy1
Emx1-NuTRAP - Triple Stain
SSC-A
FSC-A
Live 38.1
Live 4.69
b
Gate 2: Single Cell
FSC-H
FSC-A
Single Cells 68.5
Single Cells 65.6
c
Gate 3: Autofluorescence
Comp-S100B-A
Comp-Thy1-A
S100, Thy1 autofluorescence 0.94
S100, Thy1 True 99.0
S100, Thy1 autofluorescence 8.45
S100, Thy1 True 91.5
d
Gate 4a: Thy1-GFP
e
Gate 4b: S100B-GFP
Fluorescence Minus One (FMO) - Thy1
Fluorescence Minus One (FMO) - S100B
Fluorescence Minus One (FMO) - GFP
Comp-Thy1-A
Comp-S100B-A
Comp-GFP-A

Supplementary Figure 1:

Fluorescence activated cell sorting (FACS) gating strategy. **a-c** Hippocampal cell isolates from Wild Type +Thy1 (C57Bl6/J mice single stained with Thy1 AlexaFluor™ 647 antibody) and *Emx1*-NuTRAP Triple Stain (*Emx1*-NuTRAP mice expressing EGFP and stained with Thy1 antibody conjugated to AlexaFluor™ 647 and S100β antibody with an AlexaFluor™ 405 goat anti-rabbit IgG secondary antibody) were used to set Gate 1, 2 and 3. **a** Gate 1 for live cell gating was determined by analyzing forward scatter area (FSC-A) and side scatter area (SSC-A). Cells within this gate were positively sorted for Gate 2. **b** Gate 2 for single cell gating was determined by analyzing FSC-A and forward scatter height (FSC-H). Cells within this gate were positively sorted for Gate 3. **c** Gate 3 for Thy1+/S100β+ autofluorescence was determined by analyzing Comp-BV421-A (Comp-S100β-A) and Comp-Alexa Fluor 647-A (Comp-Thy1-A). Cells within this gate were negatively sorted for Gate 4. **d-e** Hippocampal cell isolates from Fluorescence Minus One (FMO) – Thy1 (*Emx1*-NuTRAP mice expressing EGFP and stained with Thy1 antibody conjugated to AlexaFluor™ 647), FMO - S100β (*Emx1*-NuTRAP mice expressing EGFP and stained with S100β antibody with an AlexaFluor™ 405 goat anti-rabbit IgG secondary antibody), FMO - GFP (C57Bl6/J mice single stained with Thy1 AlexaFluor™ 647 antibody and S100β antibody with an AlexaFluor™ 405 goat anti-rabbit IgG secondary antibody), Wild Type +Thy1 and *Emx1*-NuTRAP Triple Stain were used to set Gate 4. **d** Gate 4a for Thy1 and GFP gating was determined by analyzing Comp-GFP-A and Comp-Thy1-A. **e** Gate 4b for S100β and GFP gating was determined by analyzing Comp-GFP-A and Comp-S100β-A.

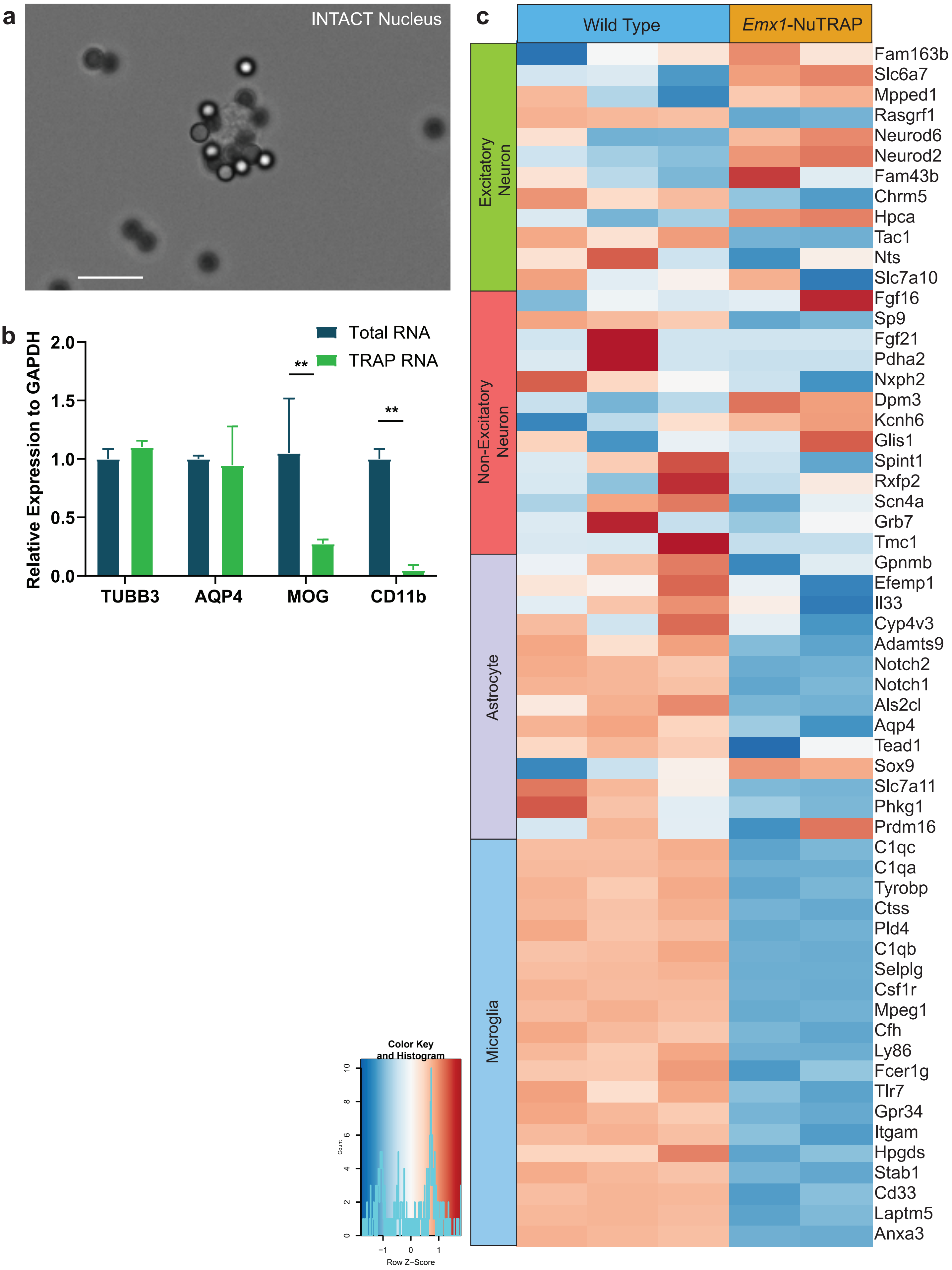
a
INTACT Nucleus
b
Total RNA
TRAP RNA
Relative Expression to GAPDH
2.0
1.5
1.0
0.5
0.0
**
**
TUBB3
AQP4
MOG
CD11b
c
Wild Type
Emx1-NuTRAP
Excitatory Neuron
Non-Excitatory Neuron
Astrocyte
Microglia
Fam163b
Slc6a7
Mpped1
Rasgrf1
Neurod6
Neurod2
Fam43b
Chrm5
Hpca
Tac1
Nts
Slc7a10
Fgf16
Sp9
Fgf21
Pdha2
Nxph2
Dpm3
Kcnh6
Glis1
Spint1
Rxfp2
Scn4a
Grb7
Tmc1
Gpnmb
Efemp1
Il33
Cyp4v3
Adamts9
Notch2
Notch1
Als2cl
Aqp4
Tead1
Sox9
Slc7a11
Phkg1
Prdm16
C1qc
C1qa
Tyrobp
Ctss
Pld4
C1qb
Selplg
Csf1r
Mpeg1
Cfh
Ly86
Fcer1g
Tlr7
Gpr34
Itgam
Hpgds
Stab1
Cd33
Laptm5
Anxa3
Color Key and Histogram
Count
Row Z-Score
-1
0
1

Supplementary Figure 2:

a Brightfield microscopy of a neural nucleus bound to streptavidin coated Dynabeads™. The scale bar represents 10µm. **b** qPCR of TRAP isolated RNA compared to total RNA from the simultaneous isolation protocol $^{**}p<0.01$. Bars represent standard error. **c** Expanded heatmap of differentially expressed genes of cell type markers for excitatory neurons, non-excitatory neurons, astrocytes, and microglia[1] from RNA-seq data comparing TRAP-isolated RNA from hippocampus of *Emx1*-NuTRAP mice vs hippocampal mRNA isolated from wild type mice.

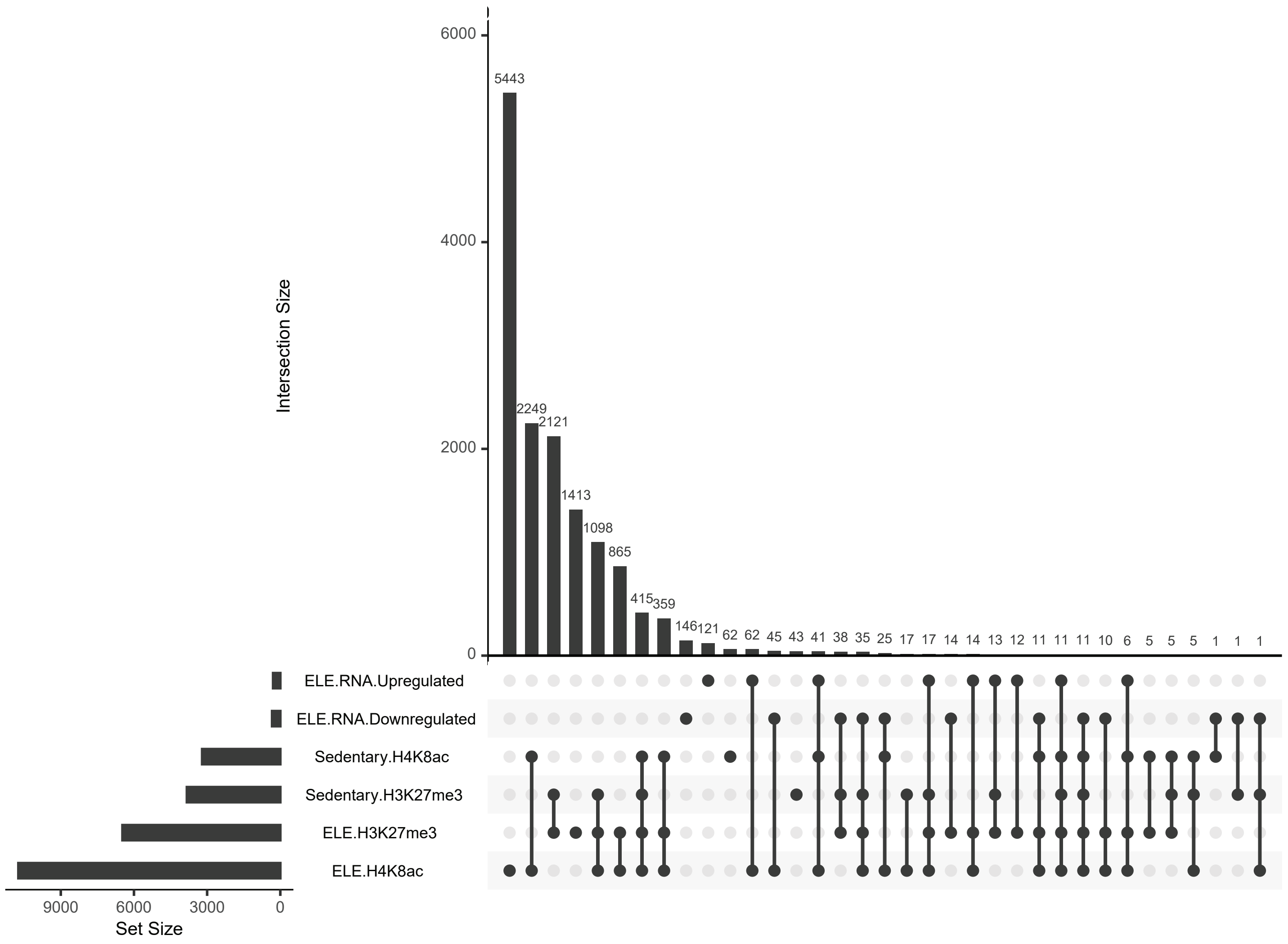

Intersection Size
6000
4000
2000
0
5443
2249
2121
1413
1098
865
415
359
146
121
62
62
45
43
41
38
35
25
17
17
14
14
13
12
11
11
11
10
6
5
5
5
1
1
1
ELE.RNA.Upregulated
ELE.RNA.Downregulated
Sedentary.H4K8ac
Sedentary.H3K27me3
ELE.H3K27me3
ELE.H4K8ac
9000
6000
3000
0
Set Size

Supplementary Figure 3:

Upset plot demonstrating the overlaps between upregulated and downregulated genes and their associations with H4K8ac and H3K27me3.

Supplementary References

1 Glock, C. *et al*. The translatome of neuronal cell bodies, dendrites, and axons. *Proc Natl Acad Sci U S A* **118**, doi:10.1073/pnas.2113929118 (2021).
