# Supplementary material for: Early-life exercise primes the murine neural epigenome to facilitate gene expression and hippocampal memory consolidation
Source: Commun Biol. 2023 Jan 7;6:18. doi: 10.1038/s42003-022-04393-7 (PMC9825372; doi:10.1038/s42003-022-04393-7)
Supplement: Supplementary file 3 — Description of Additional Supplementary Data [file 42003_2022_4393_MOESM3_ESM.pdf]

Description of Additional Supplementary Files

File name: Supplementary Data 1

Description: FACS data output and source data for Figure 1e, associated with Supplementary Figure 1.

File name: Supplementary Data 2

Description: Sample quality control data (concentration, integrity and size) for all TRAP-isolated RNA and CUT&RUN-Seq libraries.

File name: Supplementary Data 3

Description: Source data for Figure 2f-i.

File name: Supplementary Data 4

Description: Source data for Figure 3a-d.

File name: Supplementary Data 5

Description: 2-way ANOVA and Likelihood Ratio Test results

File name: Supplementary Data 6

Description: Ingenuity Pathway Analysis (IPA) results for genes upregulated after ELE and source data for Figure 4c.

File name: Supplementary Data 7

Description: Gene Set Enrichment Analysis (GSEA) results and source data for Figure 4d.

File name: Supplementary Data 8

Description: CUT&RUN-Seq histone peak calls and source data for Figure 5b.

File name: Supplementary Data 9

Description: Source data for Figure 6b and c.

File name: Supplementary Data 10

Description: Source data for Figure 6b’ and c’.

File name: Supplementary Data 11

Description: Ingenuity Pathway Analysis (IPA) results and source data for Figure 6e.
