# Supplementary material for: Early-life exercise primes the murine neural epigenome to facilitate gene expression and hippocampal memory consolidation
Source: Commun Biol. 2023 Jan 7;6:18. doi: 10.1038/s42003-022-04393-7 (PMC9825372; doi:10.1038/s42003-022-04393-7)
Supplement: Supplementary file 15 — nr-reporting-summary [file 42003_2022_4393_MOESM15_ESM.pdf]

nature portfolio

Corresponding author(s): Autumn S. Ivy

Last updated by author(s): Jul 14, 2022

Reporting Summary

Nature Portfolio wishes to improve the reproducibility of the work that we publish. This form provides structure for consistency and transparency in reporting. For further information on Nature Portfolio policies, see our Editorial Policies and the Editorial Policy Checklist.

Statistics

For all statistical analyses, confirm that the following items are present in the figure legend, table legend, main text, or Methods section.

n/a	Confirmed	
☐	☒	The exact sample size (*n*) for each experimental group/condition, given as a discrete number and unit of measurement
☐	☒	A statement on whether measurements were taken from distinct samples or whether the same sample was measured repeatedly
☐	☒	The statistical test(s) used AND whether they are one- or two-sided<br>*Only common tests should be described solely by name; describe more complex techniques in the Methods section.*
☒	☐	A description of all covariates tested
☐	☒	A description of any assumptions or corrections, such as tests of normality and adjustment for multiple comparisons
☐	☒	A full description of the statistical parameters including central tendency (e.g. means) or other basic estimates (e.g. regression coefficient) AND variation (e.g. standard deviation) or associated estimates of uncertainty (e.g. confidence intervals)
☐	☒	For null hypothesis testing, the test statistic (e.g. *F*, *t*, *r*) with confidence intervals, effect sizes, degrees of freedom and *P* value noted<br>*Give P values as exact values whenever suitable.*
☒	☐	For Bayesian analysis, information on the choice of priors and Markov chain Monte Carlo settings
☒	☐	For hierarchical and complex designs, identification of the appropriate level for tests and full reporting of outcomes
☐	☒	Estimates of effect sizes (e.g. Cohen's *d*, Pearson's *r*), indicating how they were calculated

Our web collection on statistics for biologists contains articles on many of the points above.

Software and code

Policy information about availability of computer code

Data collection	Novaseq control software version 1.7.5 and Real time analysis version 3.4.4 were used by the Genomics High Throughput Facility at UC Irvine for data collection and demultiplexing.
Data analysis	RNA-seq analysis: FastQ files were quality checked for sequencing errors using FastQC (version 0.11.9). No files were found to have sufficient quality errors to discount their use. Files were aligned using STAR Aligner (version 2.7.3a). Duplicate reads were removed using Picard Tools (version 1.87). SAM Tools was used to convert BAM files to SAM files for use in downstream analysis. FastQC, alignment and duplicate removal were all preformed on the High-Powered Compute Cluster (HPC3) operated by The Research Cyberinfrastructure Center (RCIC) at the University of California, Irvine. R (version 4.1.0) was used for differentially expressed genes (DEG) analysis. Genomic Alignments (version 1.28.0) (summarizeOverlaps mode="IntersectionNotEmpty", singleEnd=FALSE, ignore.strand=FALSE, fragments=TRUE), Genomic Features (version 1.44.0) (exons by gene), and R SAM Tools (version 2.8.0) (yieldSize=100000) were used to extract a count matrix and generate a summarized experiment object. DEseq2 (version 1.32.0) was used to perform a DEG analysis. Ensemble IDs were converted to gene symbols using BiomaRt (version 2.48.1). PCA plots using the top 2000 genes were generated using ggplot2 (version 3.3.4). Samples were determined as outliers if the variability between the samples heavily weighted PC1 to those samples. This excluded 2 sedentary samples and 1 ELE sample. Heatmaps were generated using gplots (version 3.1.1) and RColorBrewer (version1.1-2). Volcano Plots were generated using ggplot2. Venn diagrams were generated using the Venn diagram tool from Bioinformatics and Evolutionary Genomics. Gene ontology analysis was performed using Panther Classification System (version 16.0). Genes were also categorized and a leading edge heatmap was also generated using Gene Set Enrichment Analysis (version GSEA 4.1.0). Upstream regulators were identified from the DEGs upregulated by ELE using Qiagen's Ingenuity Pathway Analysis (IPA) (Fall 2021 Release).<br>CUT&RUN-seq analysis: FastQ files were quality checked for sequencing errors using FastQC (version 0.11.9). No files were found to have sufficient quality errors to discount their use. FastQ files were aligned using Bowtie2 (version 2.4.1). Significant peaks were called from these aligned files using SEACR, developed in CUT&Tag for efficient epigenomic profiling of small samples and single cells , according to CUT&Tag

Data Processing and Analysis Tutorial (updated August 12 2020): https://www.protocols.io/view/cut-amp-tag-data-processing-and-analysis-tutorial-bjk2kky. An FDR higher than 0.1 was considered too high for called peaks. This threshold excluded 4 samples (3 ELE H4K8ac separate isolation samples and 1 ELE H4K8ac SIT sample). Peaks were called using the stringent and 0.01 settings. Significant peaks were annotated using ChIPseeker (version 1.8.6) using TxDb.Mmusculus.UCSC.mm10.knownGene as a reference with the following settings: tssRegion = c(-3000, 3000), TxDb = TxDb.Mmusculus.UCSC.mm10.knownGene, level = "transcript", assignGenomicAnnotation = TRUE, genomicAnnotationPriority = c("Promoter", "5UTR", "3UTR", "Exon", "Intron", "Downstream", "Intergenic"), annoDb = NULL, addFlankGeneInfo = FALSE, flankDistance = 5000, sameStrand = FALSE, ignoreOverlap = FALSE, ignoreUpstream = FALSE, ignoreDownstream = FALSE, overlap = "TSS", verbose = TRUE). This approach annotated peaks to the closest gene by distance from the promoter, except if that peak falls within a gene before the distal intergenic region. Ensemble IDs were converted to gene symbols using BiomaRt . Gene lists of peaks present in each condition were used to generate Venn diagrams, as with RNA-seq analysis. Further Venn diagrams were generated comparing DEGs with peak calls. Peaks were visualized using UCSC Genome Browser and Genome Browser in a Box.

For manuscripts utilizing custom algorithms or software that are central to the research but not yet described in published literature, software must be made available to editors and reviewers. We strongly encourage code deposition in a community repository (e.g. GitHub). See the Nature Portfolio guidelines for submitting code & software for further information.

Data

Policy information about availability of data

All manuscripts must include a data availability statement. This statement should provide the following information, where applicable:

- Accession codes, unique identifiers, or web links for publicly available datasets
- A description of any restrictions on data availability
- For clinical datasets or third party data, please ensure that the statement adheres to our policy

Further information and requests for resources and reagents should be directed to and will be fulfilled by the lead contact, Dr. Autumn Ivy. RNA-seq and CUT&RUN-Has been deposited on to the Gene Expression Omnibus database service. The GEO SuperSeries accession number to access all data used in this study is GSE208715. This data will be make public on the date of publication. Reviewers may access this information through a reviewer token: mdefsqiythubbyv. Any additional information required to reanalyze the data reported in this paper is available from the lead contact upon request.

Human research participants

Policy information about studies involving human research participants and Sex and Gender in Research.

Reporting on sex and gender

Use the terms sex (biological attribute) and gender (shaped by social and cultural circumstances) carefully in order to avoid confusing both terms. Indicate if findings apply to only one sex or gender; describe whether sex and gender were considered in study design whether sex and/or gender was determined based on self-reporting or assigned and methods used. Provide in the source data disaggregated sex and gender data where this information has been collected, and consent has been obtained for sharing of individual-level data; provide overall numbers in this Reporting Summary. Please state if this information has not been collected. Report sex- and gender-based analyses where performed, justify reasons for lack of sex- and gender-based analysis.

Population characteristics

Describe the covariate-relevant population characteristics of the human research participants (e.g. age, genotypic information, past and current diagnosis and treatment categories). If you filled out the behavioural & social sciences study design questions and have nothing to add here, write "See above."

Recruitment

Describe how participants were recruited. Outline any potential self-selection bias or other biases that may be present and how these are likely to impact results.

Ethics oversight

Identify the organization(s) that approved the study protocol.

Note that full information on the approval of the study protocol must also be provided in the manuscript.

Field-specific reporting

Please select the one below that is the best fit for your research. If you are not sure, read the appropriate sections before making your selection.

☒ Life sciences ☐ Behavioural & social sciences ☐ Ecological, evolutionary & environmental sciences

For a reference copy of the document with all sections, see nature.com/documents/nr-reporting-summary-flat.pdf

Life sciences study design

All studies must disclose on these points even when the disclosure is negative.

Sample size

For all sequencing experiments in this study, n=2-4 mice per group, which is generally acceptable in the field. For the study of enrichment of neural markers from TRAP-seq mRNA isolation, n=2-3 mice per group. For the study on the epigenetic and transcriptional effect of exercise we started with n=3-4 mice per group, but after outlier removal, n=2-3 mice per group.

Data exclusions	Two of the samples in one of our sedentary group for the epigenetic and translating mRNA study had a high amount of ribosomal and non mRNA present in the sequencing counts. We excluded these since our isolation should have removed those and it likely means there was an error in the isolation of the RNA making analysis of the samples questionable. We removed one animal from the exercised group because it was vastly different on PCA analysis than the other three.
Replication	It would not be reasonable to repeat the study ourselves given budget and time limitations.
Randomization	We used a random number generator in Microsoft Excel to randomize samples at steps that required randomization such as library preparation.
Blinding	Blinding was not relevant to this study since all steps of the study were analyzed computationally rather than by experimenter derived metric.

Reporting for specific materials, systems and methods

We require information from authors about some types of materials, experimental systems and methods used in many studies. Here, indicate whether each material, system or method listed is relevant to your study. If you are not sure if a list item applies to your research, read the appropriate section before selecting a response.

Materials & experimental systems

n/a	Involved in the study	
☐	☒	Antibodies
☒	☐	Eukaryotic cell lines
☒	☐	Palaeontology and archaeology
☐	☒	Animals and other organisms
☒	☐	Clinical data
☒	☐	Dual use research of concern

Methods

n/a	Involved in the study	
☐	☒	ChIP-seq
☐	☒	Flow cytometry
☒	☐	MRI-based neuroimaging

Antibodies

Antibodies used	THY-1 (OX7) AlexaFluor™ 647 (Santa Cruz Biotechnology, sc-53116 AF647, Lot: F2716), S100β (Abcam, ab41548, Lot:GR3326165-1), AlexaFluor™ 405 goat anti-rabbit IgG (H+L) (Invitrogen, A31556, Lot: 2273716) secondary antibody, anti-H3K27me3 (Cell Signaling Technologies, C36B11, Lot: 16), anti-H4K8ac (Epicypher, 13-0047, Lot: 20202001-11), and anti-IgG control (Rabbit IgG Fisher Scientific, 026102)).
Validation	The validation of the manufacturers for each antibody was taken as sufficient.

Animals and other research organisms

Policy information about studies involving animals; ARRIVE guidelines recommended for reporting animal research, and Sex and Gender in Research

Laboratory animals	Emx1-IRES-Cre knock-in mice (Jackson Laboratory Stock No: 005628), NuTRAP mice (Jackson Laboratory Stock No: 029899), and C57Bl6/J (Jackson Laboratory Stock No: 000664) were obtained from Jackson Laboratories
Wild animals	none
Reporting on sex	Only male mice were used, and this is reported in the manuscript
Field-collected samples	none
Ethics oversight	Experiments were conducted according to US National Institutes of Health guidelines for animal care and use and were approved by the Institutional Animal Care and Use Committee of the University of California, Irvine.

Note that full information on the approval of the study protocol must also be provided in the manuscript.

ChIP-seq

Data deposition

☒ Confirm that both raw and final processed data have been deposited in a public database such as GEO.

☒ Confirm that you have deposited or provided access to graph files (e.g. BED files) for the called peaks.

Data access links<br>*May remain private before publication.*	The GEO SuperSeries accession number to access all data used in this study is GSE208715. This data will be make public on the date of publication. Reviewers may access this information through a reviewer token: mdefsqiythubbyv . Also we

performed CUT&RUN-seq rather than standard ChIP-seq. Here is a link to the entry: https://urldefense.com/v3/__https:/
www.ncbi.nlm.nih.gov/geo/query/acc.cgi?acc=GSE208715__;!!CzAuKJ42GuquVTTmVmPViYEvSg!
KJI133I6sT46qvCseatLSk7sON_KDXyI548XlMzI_I4-i4YG_Hlrbmw1Y1eADFkYhcFOnKBL9gCvOyM$.

Files in database submission

allH3k27me3sedsim.csv,allH3k27me3sedsim.csv,allH3k27me3exright.csv,allH3k27me3sedleft.csv,allH3k27me3sedright.csv,al
lH4K8acsedsim.csv,allH4K8acsedsim.csv,allH4K8acexright.csv,allH4K8acsedleft.csv,allH4K8acsedright.csv,allH3k27me3sedsim
.csv,allH3k27me3sedsim.csv,allH4K8acexright.csv,allH3k27me3sedleft.csv,allH4K8acsedright.csv,allH3k27me3exsim.csv,allH3
k27me3exsim.csv,allH3k27me3exsim.csv,allH3k27me3sedsim.csv,allH3k27me3exleft.csv,allH3k27me3exleft.csv,allH3k27me3
sedright.csv,allH3k27me3sedright.csv,allH4K8acexsim.csv,allH4K8acexsim.csv,allH4K8acexsim.csv,allH4K8acsedsim.csv,allH4K
8acexleft.csv,allH4K8acexleft.csv,allH4K8acsedright.csv,allH4K8acsedright.csv,allH3k27me3exsim.csv,allH3k27me3exsim.csv,
allH3k27me3exsim.csv,allH3k27me3sedsim.csv,allH3k27me3exleft.csv,allH3k27me3exleft.csv,allH4K8acsedright.csv,allH4K8a
csedright.csv,allH3k27me3exsim.csv,allH3k27me3sedsim.csv,allH3k27me3exleft.csv,allH3k27me3exright.csv,allH3k27me3exr
ight.csv,allH3k27me3sedleft.csv,allH3k27me3sedleft.csv,allH4K8acexsim.csv,allH4K8acsedsim.csv,allH4K8acexleft.csv,allH4K8
acexright.csv,allH4K8acexright.csv,allH4K8acsedleft.csv,allH4K8acsedleft.csv,allH3k27me3exsim.csv,allH3k27me3sedsim.csv,a
llH3k27me3exleft.csv,allH4K8acexright.csv,allH4K8acexright.csv,allH3k27me3sedleft.csv,allH3k27me3sedleft.csv,nR116-L4-
P041-GATGCCGG-TCGTAGAT_sortedbyreadid.bw,nR116-L4-P042-GAAGCACA-GCCAGAGG_sortedbyreadid.bw,nR116-L4-
P043-GAATATCC-ATGGCGTC_sortedbyreadid.bw,nR116-L4-P044-TCGAAGCT-CTCAGTGC_sortedbyreadid.bw,nR116-L4-P045-
TCACCAAT-GCGCCGTG_sortedbyreadid.bw,nR116-L4-P046-TGGTCATT-CCATCTGC_sortedbyreadid.bw,nR116-L4-P047-
CAGAAGAT-CAATGTAC_sortedbyreadid.bw,nR116-L4-P048-CAATCGAA-GCGTGTGC_sortedbyreadid.bw,nR116-L4-P049-
CTACGAAG-CTGTCGAG_sortedbyreadid.bw,nR116-L4-P050-CTTAATAC-CACAATGG_sortedbyreadid.bw,nR116-L4-P041-
GATGCCGG-TCGTAGAT-READ1-Sequences.txt.gz,nR116-L4-P042-GAAGCACA-GCCAGAGG-READ1-Sequences.txt.gz,nR116-L4-
P043-GAATATCC-ATGGCGTC-READ1-Sequences.txt.gz,nR116-L4-P044-TCGAAGCT-CTCAGTGC-READ1-
Sequences.txt.gz,nR116-L4-P045-TCACCAAT-GCGCCGTG-READ1-Sequences.txt.gz,nR116-L4-P046-TGGTCATT-CCATCTGC-
READ1-Sequences.txt.gz,nR116-L4-P047-CAGAAGAT-CAATGTAC-READ1-Sequences.txt.gz,nR116-L4-P048-CAATCGAA-
GCGTGTGC-READ1-Sequences.txt.gz,nR116-L4-P049-CTACGAAG-CTGTCGAG-READ1-Sequences.txt.gz,nR116-L4-P050-
CTTAATAC-CACAATGG-READ1-Sequences.txt.gz,nR116-L4-P051-CTTATGAA-ACATTGGC-READ1-Sequences.txt.gz,nR116-L4-
P052-CTATCATT-AGGTGGTC-READ1-Sequences.txt.gz,nR116-L4-P053-CTGGAAGC-TCTGCACC-READ1-Sequences.txt.gz,nR116-
L4-P054-CAACCGTG-CGTGAGAG-READ1-Sequences.txt.gz,nR116-L4-P055-TGAGGCGC-TGGTCTCC-READ1-
Sequences.txt.gz,nR116-L4-P056-AAGTACAG-GACGTGAC-READ1-Sequences.txt.gz,nR116-L4-P057-GATGCGTC-GCAGCCTC-
READ1-Sequences.txt.gz,nR116-L4-P058-GAAGTCTT-ACCGTAGT-READ1-Sequences.txt.gz,nR116-L4-P059-TCGGCACC-
GACGTTGG-READ1-Sequences.txt.gz,nR116-L4-P060-CGCGCCAA-CCTAGCCA-READ1-Sequences.txt.gz,nR116-L4-P061-
CGTAAGAG-GTCAGCGA-READ1-Sequences.txt.gz,nR116-L4-P062-TGGTGCAC-GTGATTCC-READ1-Sequences.txt.gz,nR116-L4-
P063-CAGGTTCC-GGTGTTCG-READ1-Sequences.txt.gz,nR116-L4-P064-CCGTGCCA-GGAGCTGC-READ1-
Sequences.txt.gz,nR116-L4-P065-CTACGGCA-CTACGAGG-READ1-Sequences.txt.gz,nR116-L4-P066-GAAGAGGC-TAGCACTT-
READ1-Sequences.txt.gz,nR116-L4-P067-AAGAAGCG-TTATCTAC-READ1-Sequences.txt.gz,nR116-L4-P068-CGAACGGA-
CCGGTAGG-READ1-Sequences.txt.gz,nR116-L4-P069-AAGAGAGC-ACTGGCTG-READ1-Sequences.txt.gz,nR116-L4-P070-
CCACAATG-CGTCAACG-READ1-Sequences.txt.gz,nR116-L4-P071-TGAACAGG-TTGTTCTC-READ1-Sequences.txt.gz,nR116-L4-
P072-CATTGCAC-GCTGCATG-READ1-Sequences.txt.gz,nR116-L4-P073-GATTCCTT-TGCCTATG-READ1-Sequences.txt.gz,nR116-
L4-P074-CGGATAAC-CGTTCGGC-READ1-Sequences.txt.gz,nR116-L4-P075-CTCACGAT-GCCGTAAG-READ1-
Sequences.txt.gz,nR116-L4-P076-CTACTGAC-CATCCTCC-READ1-Sequences.txt.gz,nR116-L4-P077-TCAACGAG-GGTTGACG-
READ1-Sequences.txt.gz,nR116-L4-P078-TGTGTGCC-ACCTGATG-READ1-Sequences.txt.gz,nR116-L4-P079-CAGTGTGG-
GATTCACA-READ1-Sequences.txt.gz,nR116-L4-P080-TTACCTGG-GCAATCGT-READ1-Sequences.txt.gz,nR116-L4-P081-
CTACTCGA-GTTCATCT-READ1-Sequences.txt.gz,nR116-L4-P082-GAATCTGG-CGTGGACA-READ1-Sequences.txt.gz,nR116-L4-
P083-TCGGTCGA-GCACTTGG-READ1-Sequences.txt.gz,nR116-L4-P084-TCTTAAGT-ATTCAGCG-READ1-
Sequences.txt.gz,nR116-L4-P085-TGCGAGAC-TCGCCGGC-READ1-Sequences.txt.gz,nR116-L4-P086-CACCACGG-TTACAACG-
READ1-Sequences.txt.gz,nR116-L4-P087-TTCCACCA-CCTTAACC-READ1-Sequences.txt.gz,nR116-L4-P088-CTGCAACG-
CGAATGTC-READ1-Sequences.txt.gz,nR116-L4-P089-GATTCGAG-ATACTCGG-READ1-Sequences.txt.gz,nR116-L4-P090-
CTGATTGA-AGTGTGTC-READ1-Sequences.txt.gz,nR116-L4-P091-TCGGTAAG-TGAGTTAG-READ1-Sequences.txt.gz,nR116-L4-
P092-TTAGAGTC-TCGTCACA-READ1-Sequences.txt.gz,nR116-L4-P093-CCTGGTGT-CCGGAGGC-READ1-
Sequences.txt.gz,nR116-L4-P094-TGTGTTAA-ACCGTGCC-READ1-Sequences.txt.gz,nR116-L4-P095-CCGCTGTT-TGCGGTCT-
READ1-Sequences.txt.gz,nR116-L4-P096-TACTGTTA-GTATTCGC-READ1-Sequences.txt.gz,nR116-L4-P097-TAGCCGAT-
AGTATTGG-READ1-Sequences.txt.gz,nR116-L4-P098-CGATCCAC-ATGCGCGA-READ1-Sequences.txt.gz,nR116-L4-P099-
TAGCGTTG-GTTCACAC-READ1-Sequences.txt.gz,nR116-L4-P100-CTCATCAC-GTGCCACC-READ1-Sequences.txt.gz,nR116-L4-
P041-GATGCCGG-TCGTAGAT-READ2-Sequences.txt.gz,nR116-L4-P042-GAAGCACA-GCCAGAGG-READ2-
Sequences.txt.gz,nR116-L4-P043-GAATATCC-ATGGCGTC-READ2-Sequences.txt.gz,nR116-L4-P044-TCGAAGCT-CTCAGTGC-
READ2-Sequences.txt.gz,nR116-L4-P045-TCACCAAT-GCGCCGTG-READ2-Sequences.txt.gz,nR116-L4-P046-TGGTCATT-
CCATCTGC-READ2-Sequences.txt.gz,nR116-L4-P047-CAGAAGAT-CAATGTAC-READ2-Sequences.txt.gz,nR116-L4-P048-
CAATCGAA-GCGTGTGC-READ2-Sequences.txt.gz,nR116-L4-P049-CTACGAAG-CTGTCGAG-READ2-Sequences.txt.gz,nR116-L4-
P050-CTTAATAC-CACAATGG-READ2-Sequences.txt.gz,nR116-L4-P051-CTTATGAA-ACATTGGC-READ2-Sequences.txt.gz,nR116-
L4-P052-CTATCATT-AGGTGGTC-READ2-Sequences.txt.gz,nR116-L4-P053-CTGGAAGC-TCTGCACC-READ2-
Sequences.txt.gz,nR116-L4-P054-CAACCGTG-CGTGAGAG-READ2-Sequences.txt.gz,nR116-L4-P055-TGAGGCGC-TGGTCTCC-
READ2-Sequences.txt.gz,nR116-L4-P056-AAGTACAG-GACGTGAC-READ2-Sequences.txt.gz,nR116-L4-P057-GATGCGTC-
GCAGCCTC-READ2-Sequences.txt.gz,nR116-L4-P058-GAAGTCTT-ACCGTAGT-READ2-Sequences.txt.gz,nR116-L4-P059-
TCGGCACC-GACGTTGG-READ2-Sequences.txt.gz,nR116-L4-P060-CGCGCCAA-CCTAGCCA-READ2-Sequences.txt.gz,nR116-L4-
P061-CGTAAGAG-GTCAGCGA-READ2-Sequences.txt.gz,nR116-L4-P062-TGGTGCAC-GTGATTCC-READ2-
Sequences.txt.gz,nR116-L4-P063-CAGGTTCC-GGTGTTCG-READ2-Sequences.txt.gz,nR116-L4-P064-CCGTGCCA-GGAGCTGC-
READ2-Sequences.txt.gz,nR116-L4-P065-CTACGGCA-CTACGAGG-READ2-Sequences.txt.gz,nR116-L4-P066-GAAGAGGC-
TAGCACTT-READ2-Sequences.txt.gz,nR116-L4-P067-AAGAAGCG-TTATCTAC-READ2-Sequences.txt.gz,nR116-L4-P068-
CGAACGGA-CCGGTAGG-READ2-Sequences.txt.gz,nR116-L4-P069-AAGAGAGC-ACTGGCTG-READ2-Sequences.txt.gz,nR116-L4-
P070-CCACAATG-CGTCAACG-READ2-Sequences.txt.gz,nR116-L4-P071-TGAACAGG-TTGTTCTC-READ2-
Sequences.txt.gz,nR116-L4-P072-CATTGCAC-GCTGCATG-READ2-Sequences.txt.gz,nR116-L4-P073-GATTCCTT-TGCCTATG-
READ2-Sequences.txt.gz,nR116-L4-P074-CGGATAAC-CGTTCGGC-READ2-Sequences.txt.gz,nR116-L4-P075-CTCACGAT-
GCCGTAAG-READ2-Sequences.txt.gz,nR116-L4-P076-CTACTGAC-CATCCTCC-READ2-Sequences.txt.gz,nR116-L4-P077-
TCAACGAG-GGTTGACG-READ2-Sequences.txt.gz,nR116-L4-P078-TGTGTGCC-ACCTGATG-READ2-Sequences.txt.gz,nR116-L4-
P079-CAGTGTGG-GATTCACA-READ2-Sequences.txt.gz,nR116-L4-P080-TTACCTGG-GCAATCGT-READ2-

Sequences.txt.gz,nR116-L4-P081-CTACTCGA-GTTCATCT-READ2-Sequences.txt.gz,nR116-L4-P082-GAATCTGG-CGTGGACA-READ2-Sequences.txt.gz,nR116-L4-P083-TCGGTCGA-GCACTTGG-READ2-Sequences.txt.gz,nR116-L4-P084-TCTTAAGT-ATTCAGCG-READ2-Sequences.txt.gz,nR116-L4-P085-TGCGAGAC-TCGCCGGC-READ2-Sequences.txt.gz,nR116-L4-P086-CACCACGG-TTACAACG-READ2-Sequences.txt.gz,nR116-L4-P087-TTCCACCA-CCTTAACC-READ2-Sequences.txt.gz,nR116-L4-P088-CTGCAACG-CGAATGTC-READ2-Sequences.txt.gz,nR116-L4-P089-GATTCGAG-ATACTCGG-READ2-Sequences.txt.gz,nR116-L4-P090-CTGATTGA-AGTGTGTC-READ2-Sequences.txt.gz,nR116-L4-P091-TCGGTAAG-TGAGTTAG-READ2-Sequences.txt.gz,nR116-L4-P092-TTAGAGTC-TCGTCACA-READ2-Sequences.txt.gz,nR116-L4-P093-CCTGGTGT-CCGGAGGC-READ2-Sequences.txt.gz,nR116-L4-P094-TGTGTTAA-ACCGTGCC-READ2-Sequences.txt.gz,nR116-L4-P095-CCGCTGTT-TGCGGTCT-READ2-Sequences.txt.gz,nR116-L4-P096-TACTGTTA-GTATTCGC-READ2-Sequences.txt.gz,nR116-L4-P097-TAGCCGAT-AGTATTGG-READ2-Sequences.txt.gz,nR116-L4-P098-CGATCCAC-ATGCGCGA-READ2-Sequences.txt.gz,nR116-L4-P099-TAGCGTTG-GTTCACAC-READ2-Sequences.txt.gz,nR116-L4-P100-CTCATCAC-GTGCCACC-READ2-Sequences.txt.gz

Genome browser session (e.g. UCSC)

not applicable

Methodology

Replicates

Only biological replicates were used and replicate agreement was verified using a PCA of counts at called peaks.

Sequencing depth

A sequencing depth of a minimum of 10 million reads was used, at least 500,000 were uniquely mapped, read length was 100bp, and the reads were paired-end.

Antibodies

anti-H3K27me3 (Cell Signaling Technologies, C36B11, Lot: 16), anti-H4K8ac (Epicypher, 13-0047, Lot: 20202001-11), and anti-IgG control (Rabbit IgG Fisher Scientific, 026102)).

Peak calling parameters

SEACR was used to call peaks. This threshold excluded 4 samples (3 ELE H4K8ac separate isolation samples and 1 ELE H4K8ac SIT sample). Peaks were called using the stringent and 0.01 settings.

Data quality

An FDR higher than 0.1 was considered too high for called peaks.

Software

Significant peaks were called from these aligned files using SEACR, developed in CUT&Tag for efficient epigenomic profiling of small samples and single cells, according to CUT&Tag Data Processing and Analysis Tutorial (updated August 12 2020): https://www.protocols.io/view/cut-amp-tag-data-processing-and-analysis-tutorial-bjk2kky. An FDR higher than 0.1 was considered too high for called peaks. This threshold excluded 4 samples (3 ELE H4K8ac separate isolation samples and 1 ELE H4K8ac SIT sample). Peaks were called using the stringent and 0.01 settings. Significant peaks were annotated using ChIPseeker (version 1.8.6) using TxDb.Mmusculus.UCSC.mm10.knownGene as a reference with the following settings: tssRegion = c(-3000, 3000), TxDb = TxDb.Mmusculus.UCSC.mm10.knownGene, level = "transcript", assignGenomicAnnotation = TRUE, genomicAnnotationPriority = c("Promoter", "5UTR", "3UTR", "Exon", "Intron", "Downstream", "Intergenic"), annoDb = NULL, addFlankGeneInfo = FALSE, flankDistance = 5000, sameStrand = FALSE, ignoreOverlap = FALSE, ignoreUpstream = FALSE, ignoreDownstream = FALSE, overlap = "TSS", verbose = TRUE). This approach annotated peaks to the closest gene by distance from the promoter, except if that peak falls within a gene before the distal intergenic region. Ensemble IDs were converted to gene symbols using BiomaRt.

Flow Cytometry

Plots

Confirm that:

☒ The axis labels state the marker and fluorochrome used (e.g. CD4-FITC).

☒ The axis scales are clearly visible. Include numbers along axes only for bottom left plot of group (a 'group' is an analysis of identical markers).

☒ All plots are contour plots with outliers or pseudocolor plots.

☒ A numerical value for number of cells or percentage (with statistics) is provided.

Methodology

Sample preparation

Fluorescence activated cell sorting (FACS) was performed to characterize neuronal and astrocytic NuTRAP cassette expression. Whole hippocampal tissue from Emx1-NuTRAP mice was isolated and single-cell suspensions were immunostained for cytometric analysis, as previously described(reference 92 in the paper). We used antibodies for THY-1 (OX7) AlexaFluor™ 647 (Santa Cruz Biotechnology, sc-53116 AF647, Lot: F2716; concentration: 1:50) and S100β (Abcam, ab41548, Lot:GR3326165-1; concentration: 1:200) with an AlexaFluor™ 405 goat anti-rabbit IgG (H+L) (Invitrogen, A31556, Lot: 2273716; concentration:1:800) secondary antibody.

Instrument

BD FACSAria™ Fusion Flow Cytometer (BD Biosciences) at the University of California, Irvine Stem Cell Core

Software

FlowJo v10.8.1 software (BD Biosciences)

Cell population abundance

15.3% of the cells were eGFP+ and Thy1+ and only 0.13% of cells were eGFP+ S100B+. This result was verified using RNA-seq on samples isolated for neurons with our described method and compared to a bulk wild type tissue from the same region of the brain.

Gating strategy

Samples and controls were positively gated for live cells (SSC-A and FSC-A) and single cells (FSC-H and FSC-A), and negatively gated for autofluorescence (Comp-Alexa Fluor 647-A (Thy1) and Comp-BV421-A (S100β)). Fraction of GFP+ neuronal cells was determined using quartile analysis of Thy-1 and Comp-GFP-A (GFP). Fraction of GFP+ astrocytic cells was determined using quartile analysis of S100β and Comp-GFP-A (GFP).

☒ Tick this box to confirm that a figure exemplifying the gating strategy is provided in the Supplementary Information.
